# SARS‐CoV‐2 vaccinations can trigger polyradiculitis

**DOI:** 10.1002/ccr3.6645

**Published:** 2022-11-23

**Authors:** Josef Finsterer, Talal Almas

**Affiliations:** ^1^ Neurology & Neurophysiology Center Vienna Austria; ^2^ Royal College of Surgeons in Ireland (RCSI) University of Medicine and Health Sciences Dublin Ireland

To the Editor,

We eagerly read the article by Aldeeb et al.^1^ about an 83‐year‐old female patient with diabetes, arterial hypertension, hyperlipidemia, and bilateral knee arthroplasty, who subsequently developed progressive quadriparesis with sensory disturbances 7 days after the first dose of the Biontech‐Pfizer anti‐SARS‐CoV‐2 vaccine. Because nerve conduction studies (NCS) were indicative of demyelinating polyneuropathy, the patient was diagnosed with Guillain–Barre syndrome (GBS), subtype acute, and inflammatory demyelinating polyneuropathy (AIDP).^1^ The diagnosis was confirmed by the good efficacy of intravenous immunoglobulins (IVIGs).^1^ GBS was attributed to the vaccination because of the temporal relation between the two, and after relevant differential causes had been ruled out.^1^ The study is appealing but raises concerns which need to be discussed.

A limitation is that the condition of the peripheral nerves before vaccination was not described. The patient had diabetes but it is not mentioned for how many years.^1^ Her HbA1c value was 6.8 on admission, but it is reported that she had numbness in her feet due to diabetes before admission. It is therefore conceivable that the patient already had polyneuropathy before onset of the GBS. We should know whether there was a difference between the results of nerve conduction studies (NCSs) before and after GBS. Since the presented NCS results could also be explained by diabetic polyneuropathy, it is crucial to know whether the NCS results were actually worse compared to previous ones.

Another limitation is that the diagnostic criteria used to fix GBS in the patient were not mentioned. The Brighton criteria are most commonly used to diagnose GBS, but sometimes the Besta, Ashford, or Hadden criteria are applied. It should be reported what level of diagnostic certainty was reached when applying the Brighton criteria.

Another limitation is that the results of cerebro‐spinal fluid (CSF) tests were not presented. Knowing whether dissociation cyto‐albuminique was present is critical to the application of all diagnostic criteria and would strengthen the diagnosis. In addition, CSF investigations could reveal whether cytokines, such as IL‐6, IL‐8, IL1a, or TNF‐alpha or glial markers, were upregulated, as has been shown in patients with SARS‐CoV‐2‐associated GBS.^2^

Since GBS can affect not only the peripheral nerves but also the cranial nerves,^3^ it should be reported whether any of the cranial nerves were affected at clinical examination or NCSs. The cranial nerve most commonly affected in GBS is the facial nerve, followed by the lower cranial nerves. Therefore, GBS patients often present with facial diplegia, dysphagia, or dysarthria.

Another limitation is that there is no mention whether there was peripheral autonomic nervous system (ANS) involvement. Because the ANS in GBS can be affected along with motor or sensory fibers or even without motor or sensory fiber involvement (pure dysautonomia),^4^ we should know whether the patient had any autonomic disturbances.

Overall, the interesting report has several limitations that should be discussed to support the conclusions. Most importantly, peripheral nerve status before onset of GBS should be reported.

## AUTHOR CONTRIBUTIONS

JF involved in design, literature search, discussion, first draft, critical comments, and final approval.

## CONFLICT OF INTEREST

None.

## ETHICAL APPROVAL

It was in accordance with ethical guidelines. The study was approved by the institutional review board.

## CONSENT

Written informed consent was obtained from the patient to publish this report in accordance with the journal's patient consent policy.

## Data Availability

All data are available from the corresponding author.

## References

[1] Aldeeb M , Okar L , Mahmud SS , Adeli GA . Could Guillain‐Barré syndrome be triggered by COVID‐19 vaccination? Clin Case Rep. 2022;10(1):e05237. doi:10.1002/ccr3.5237 35079381 PMC8765088

[2] Gigli GL , Vogrig A , Nilo A , et al. HLA and immunological features of SARS‐CoV‐2‐induced Guillain‐Barré syndrome. Neurol Sci. 2020;41(12):3391‐3394. doi:10.1007/s10072-020-04787-7 33006723 PMC7530349

[3] Finsterer J , Scorza FA , Scorza C , Fiorini A . COVID‐19 associated cranial nerve neuropathy: a systematic review. Bosn J Basic Med Sci. 2021;22:39‐45. doi:10.17305/bjbms.2021.6341 PMC8860318 34392827

[4] Biassoni E , Assini A , Gandoglia I , et al. The importance of thinking about Guillain‐Barré syndrome during the COVID‐19 pandemic: a case with pure dysautonomic presentation. J Neurovirol. 2021;27(4):662‐665. doi:10.1007/s13365-021-00997-7 34341959 PMC8328119

